# Anti-Inflammatory Benefits of Antibiotics: Tylvalosin Induces Apoptosis of Porcine Neutrophils and Macrophages, Promotes Efferocytosis, and Inhibits Pro-Inflammatory CXCL-8, IL1α, and LTB_4_ Production, While Inducing the Release of Pro-Resolving Lipoxin A_4_ and Resolvin D1

**DOI:** 10.3389/fvets.2018.00057

**Published:** 2018-04-11

**Authors:** Ruth Moges, Dimitri Desmonts De Lamache, Saman Sajedy, Bernard S. Renaux, Morley D. Hollenberg, Gregory Muench, Elizabeth M. Abbott, Andre G. Buret

**Affiliations:** ^1^Department of Biological Sciences, University of Calgary, Calgary, AB, Canada; ^2^Inflammation Research Network, University of Calgary, Calgary, AB, Canada; ^3^Department of Physiology and Pharmacology, University of Calgary, Calgary, AB, Canada; ^4^University of Calgary Veterinary Medicine, University of Calgary, Calgary, AB, Canada; ^5^ECO Animal Health, London, United Kingdom

**Keywords:** tylvalosin, macrophages, neutrophils, pig models, inflammation mediators, inflammation and its resolution, lipid mediators

## Abstract

Excessive accumulation of neutrophils and their uncontrolled death by necrosis at the site of inflammation exacerbates inflammatory responses and leads to self-amplifying tissue injury and loss of organ function, as exemplified in a variety of respiratory diseases. In homeostasis, neutrophils are inactivated by apoptosis, and non phlogistically removed by neighboring macrophages in a process known as efferocytosis, which promotes the resolution of inflammation. The present study assessed the potential anti-inflammatory and pro-resolution benefits of tylvalosin, a recently developed broad-spectrum veterinary macrolide derived from tylosin. Recent findings indicate that tylvalosin may modulate inflammation by suppressing NF-κB activation. Neutrophils and monocyte-derived macrophages were isolated from fresh blood samples obtained from 12- to 22-week-old pigs. Leukocytes exposed to vehicle or to tylvalosin (0.1, 1.0, or 10 µg/mL; 0.096–9.6 µM) were assessed at various time points for apoptosis, necrosis, efferocytosis, and changes in the production of cytokines and lipid mediators. The findings indicate that tylvalosin increases porcine neutrophil and macrophage apoptosis in a concentration- and time-dependent manner, without altering levels of necrosis or reactive oxygen species production. Importantly, tylvalosin increased the release of pro-resolving Lipoxin A_4_ (LXA_4_) and Resolvin D1 (RvD__1__) while inhibiting the production of pro-inflammatory Leukotriene B4 (LTB_4_) in Ca^2+^ ionophore-stimulated porcine neutrophils. Tylvalosin increased neutrophil phospholipase C activity, an enzyme involved in releasing arachidonic acid from membrane stores. Tylvalosin also inhibited pro-inflammatory chemokine (C–X–C motif) ligand 8 (CXCL-8, also known as Interleukin-8) and interleukin-1 alpha (IL-1α) protein secretion in bacterial lipopolysaccharide-stimulated macrophages. Together, these data illustrate that tylvalosin has potent immunomodulatory effects in porcine leukocytes in addition to its antimicrobial properties.

## Introduction

The resolution of inflammation is critical for the restoration of homeostasis following infection or tissue injury (1–4). For example, *Mycoplasma* sp. and *Actinobacillus pleuropneumoniae* colonize the lungs of swine and release leukotoxins, which in turn lyse neutrophils and macrophages (5–8). This promotes self-perpetuating inflammation (2–4, 7), as activated leukocytes release more pro-inflammatory mediators, including IL-1α, IL-1β, IL-6, CXCL-8 (synonymous with IL-8), and leukotriene B_4_ (9–12). This self-amplifying process is characteristic of the pathophysiology of pneumonia (3, 4, 9, 13).

Neutrophil apoptosis, integral to the resolution of inflammation (14, 15), is characterized by membrane blebbing, chromatic condensation, DNA fragmentation, and the formation of apoptotic bodies (16). If left uncleared, apoptotic bodies will undergo secondary necrosis (17–19). Removal of apoptotic bodies by macrophages, known as efferocytosis, plays a central role in the resolution of inflammation (17, 18, 20, 21). Efferocytosis triggers an anti-inflammatory macrophage phenotype which reduces pro-inflammatory mediators, like IL-6, LBT_4_, CXCL-8, and TNF-α, and promotes anti-inflammatory mediators, such as TGF-β, LXA^4^, and IL-10 (21–23). Pro-resolution mediators, including lipoxins and resolvins (24, 25) help resolve inflammation. Lipoxins have dual anti-inflammatory and pro-resolution properties such as increasing efferocytosis while inducing the production of anti-inflammatory IL-10, as well as decreasing pro-inflammatory cytokines and acting as analgesics (20, 24–27). This family of lipid mediators is generated *via* 15-lipoxygenase signaling from arachidonic acid, and 15(S)-HETE precursors (20, 24, 27). Docosahexaenoic acid metabolites like resolvin D1 (RvD_1_) are derived from omega-3 and 6 fatty acid synthesis (28). RvD_1_ decreases neutrophil accumulation in the lung, reduces edema, and inhibits local production of pro-inflammatory mediators (29). RvD_1_, as well as its aspirin-triggered epimer, play a central role in the self-resolution of gram-negative bacterial pneumonia (30, 31). Over the past decade, researchers both in human and veterinary medicine have been screening for therapeutics that may halt self-perpuating inflammation by stimulating anti-inflammatory and/or pro-resolution mediators (13, 32–34).

Macrolide antibiotics have broad immune-modulating properties (13, 23, 35–39). Macrolides accumulate with high affinity in phagocytes and have been shown to reach intracellular concentrations up to 500 times greater than systemic levels (35), which provides these drugs with superior pharmacodynamics (13, 40, 41). Very little is known of how macrolides may alter lipid mediator production in inflamed tissues. To address this question, we used a model of porcine cells and tylvalosin, a new broad-spectrum anti-infective veterinary macrolide derived from tylosin (39, 42). Tylvalosin’s tradename is Aivlosin^®^. Tylvalosin is a broad-spectrum third-generation macrolide derived from tylosin through the modification of 3-acetyl-4′-isovaleryl (acetylisovaleryltylosin tartrate). Tylvalosin is currently used to control respiratory and enteric bacterial infections in poultry and swine against pathogens such as *Mycoplasma* sp. (43, 44). Tylvalosin was recently found to exhibit anti-inflammatory properties by suppressing NF-κB, the transcription factor leading to the synthesis of potent pro-inflammatory mediators, such as CXCL-8 (also known as IL-8), in models of lipopolysaccharide (LPS)-induced pulmonary inflammation in mice or in piglets infected with Porcine Reproductive and Respiratory Syndrome Virus (PRRSV) (42). Using tylvalosin and porcine leukocytes as a model system, the present findings demonstrate that an antibiotic may promote neutrophil apoptosis and efferocytosis, and inhibit pro-inflammatory LTB4 production while inducing the synthesis of LXA_4_ and RvD_1_ independently of its antimicrobial actions.

## Results

### Tylvalosin Induces Concentration- and Time-Dependent Apoptosis of Porcine Neutrophils

**Figure 1:**
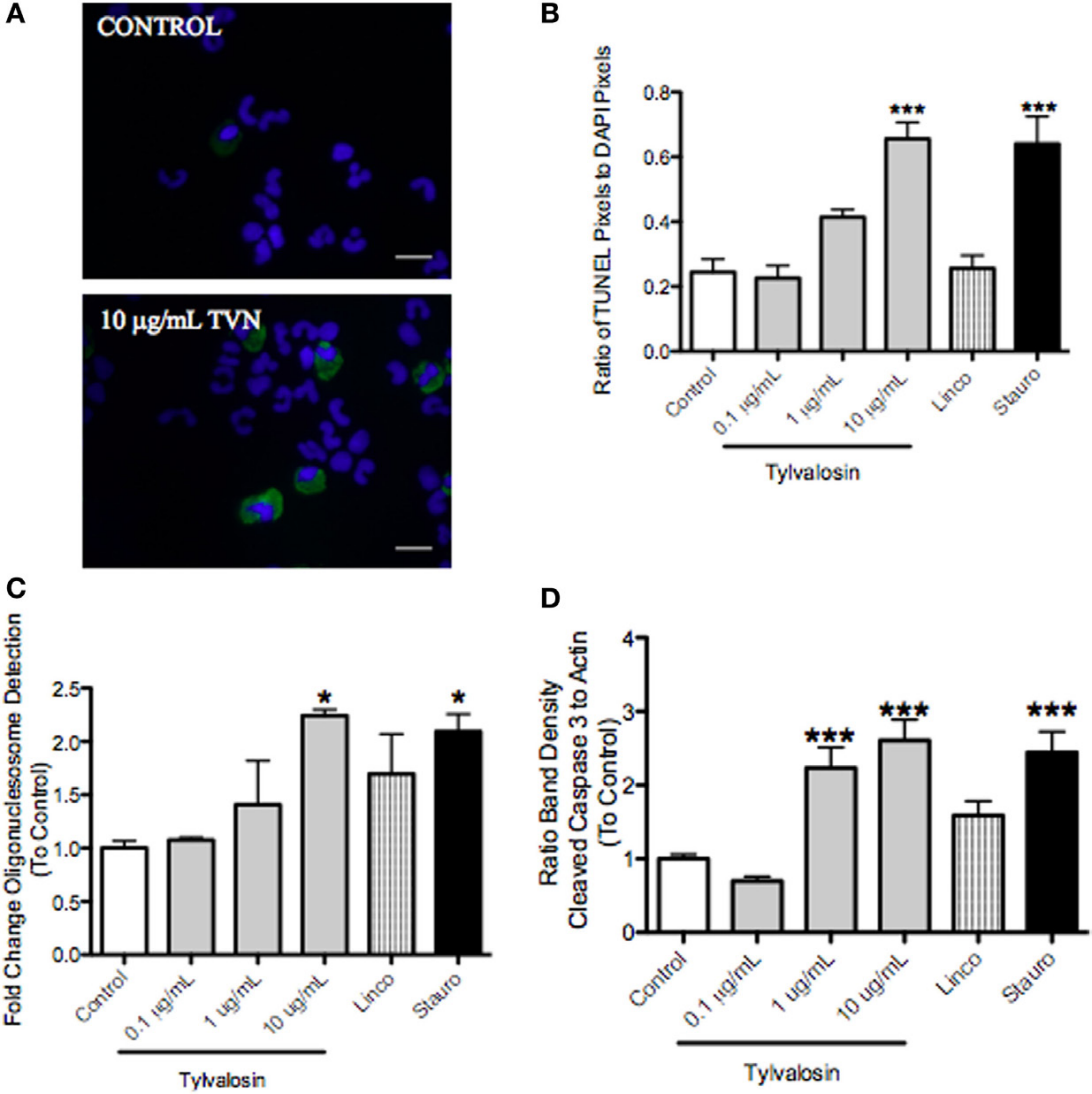
Tylvalosin induces apoptosis in porcine neutrophils. **(A)** Visual representation of fluorescent TUNEL staining of apoptotic neutrophils. Green depicts positive TUNEL staining in apoptotic cells; blue depicts DAPI-stained nuclei. Greater numbers of neutrophils positive for TUNEL (i.e. apoptosis) were found in the tylvalosin-treated group (1 h, 10 μg/mL). Magnification 1,000x. **(B)** Quantification of TUNEL staining experiments after 1-h treatment. Values were calculated as a ratio of TUNEL pixels to DAPI pixels. Data are means ± (*n* = 3–6/group) ***P < 0.001 versus control. **(C)** Cell death enzyme-linked immunosorbent assay (ELISA) showing levels of mono- and oglio-nucelosomes released during apoptotic death. Values were calculated as fold change of absorbance to control. Data are at 1 h posttreatment with control vehicle (10% FBS in HBSS), various concentrations of tylvalosin, lincomycin, and staurosporine (positive control). Data are means ± SEM (*n* = 3–4/group) *P < 0.05 versus control. **(D)** Densitometry analysis of western blot data for fragmentation of pro-apoptotic caspase-3 relative to β-actin assayed in porcine neutrophils at 0.5 h posttreatment with control (10% FBS in HBSS), TVN (0.1, 1, or 10 μg/mL), and positive control 1 μM staurosporine or equimolar concentrations (11.3 μM) of lincomycin. Values were calculated as the band density of cleaved caspase-3 (17 kDa) relative to β-actin (45 kDa), and expressed as a ratio to control. Data are expressed as group mean ± SEM (*n* = 3–6/group). ***P < 0.001 versus control.

Caspase-3 cleavage, which leads to DNA fragmentation during apoptosis, was significantly greater in tylvalosin-treated porcine neutrophils versus controls (Figure 1). Concentration- and time-dependent (0.5 and 1 h) induction of neutrophil apoptosis by tylvalosin was confirmed using *in situ* TUNEL staining, and an enzyme-linked immunosorbent assay (ELISA) detecting mono- and oligo-nucleosomes resulting from apoptosis (Figure 1). Lincomycin, another antibiotic used to treat porcine pneumonia, did not induce apoptosis. The experiments also detected the induction of apoptosis by the positive control staurosporine.

### Tylvalosin Induces Concentration- and Time-Dependent Apoptosis of Porcine Monocyte-Derived Macrophages

**Figure 2:**
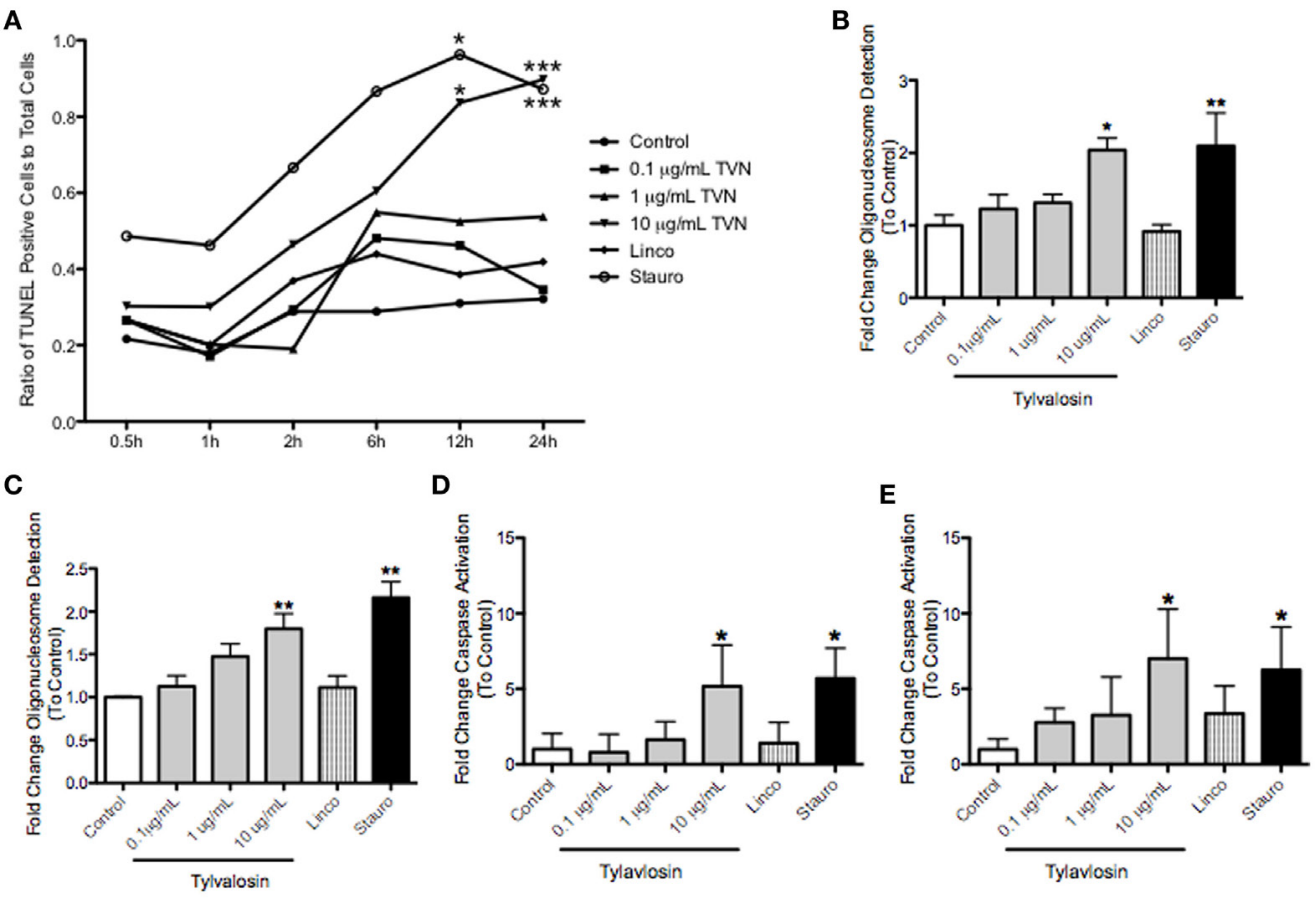
Tylvalosin induces apoptosis in porcine macrophages in a time- and dose-dependent manner. Monocyte-derived macropahges were treated with control vehicle (10% FBS in IMDM), tylvalosin (0.1, 1, or 10 μg/mL), or 1 μM staurosporine (positive control), or equimolar concentrations (11 μM) of lincomycin. **(A)** Tylvalosin-induced macrophage apoptosis is time and dose dependent. Values are reported as ratios of TUNEL stained cells in each image versus those that stained for nuclear DAPI (0.5–24 h). Data are means (*n* = 4–8/group) *P < 0.05, ***P < 0.001 versus control. Under the same conditions, a cell-death enzyme-linked immunosurbent assay (ELISA) was performed at **(B)** 12 and **(C)** 24 h. The assay is a quantitative measure of cytosolic mono- and olgio-nucleosomes after the induction of cell death. Values were calculated as fold change of absorbance to control. Data are means ± SEM (*n* = 3–5/group). *P < 0.05, **P < 0.01 versus control. Tylvalosin activates caspase-3 in porcine macrophages at 12 **(D)** and 24 h **(E)**. Data are means ± SEM (*n* = 3–5/group) *P < 0.05 versus control.

Tylvalosin, but not lincomycin, also induced time- and concentration-dependent apoptosis of porcine macrophages at 12 and 24 h posttreatment, but not at earlier time points (Figure 2). This was demonstrated using *in situ* TUNEL staining, production of mono and oligo-nucleosomes, and significantly elevated levels of cleaved caspase-3. These effects were also seen with the positive control staurosporine, but not with lincomycin.

### Tylvalosin Does Not Affect Necrosis of Porcine Neutrophils or Macrophages

**Figure 3:**
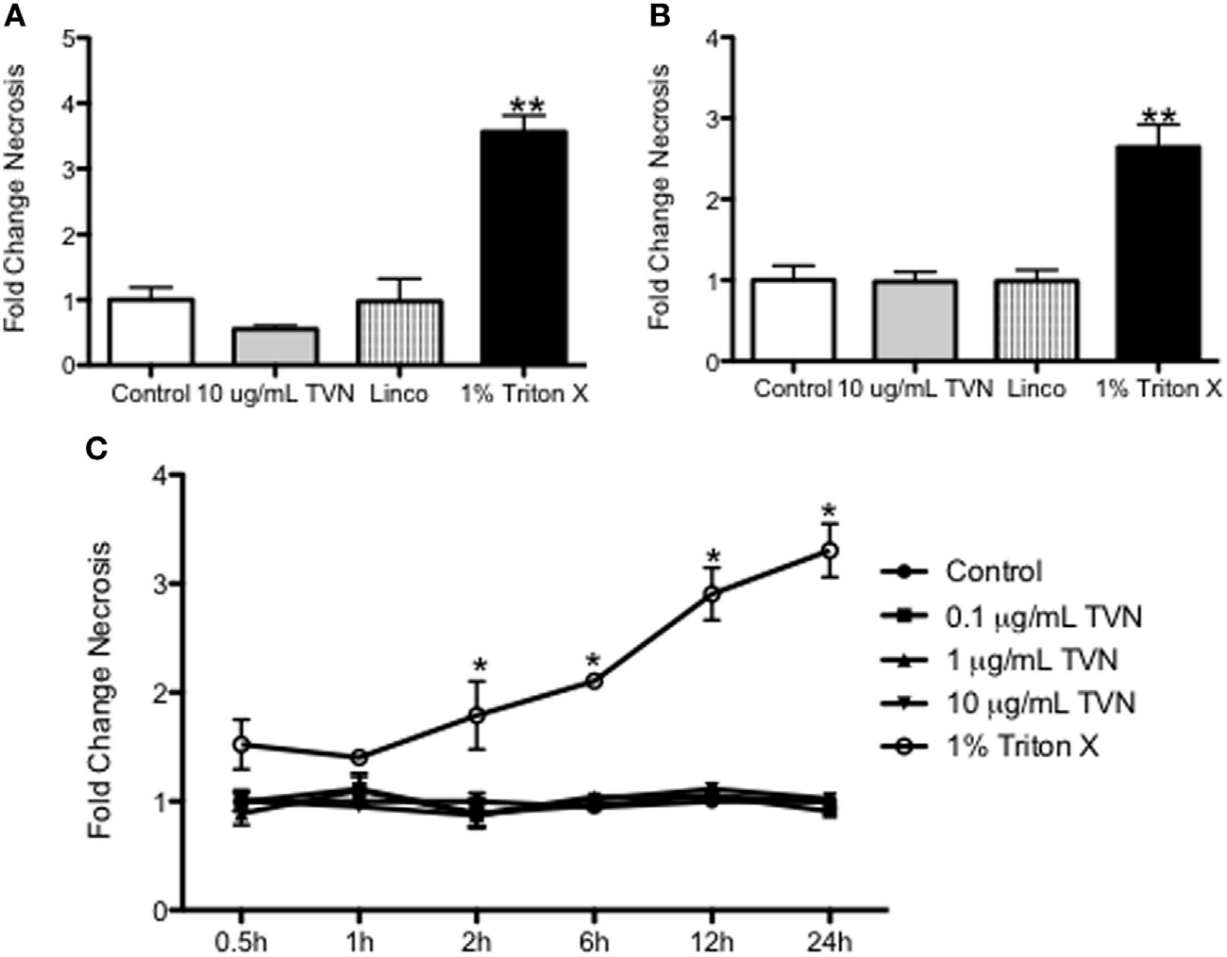
Tylvalosin has no significant effect on resting cell necrosis in vitro. Secreted lactate dehydrogenase (LDH) was measured to assess cell necrosis, by quantifying the amount of LDH protein in cell culture supernatants. Porcine neutrophils were incubated with control (10% FBS in HBSS), 10 μg/mL tylvalosin, equimolar concentrations of lincomysin (linco; 11 μM) and 1% Triton X-100 (positive control). These values were then expressed as a fold change compared to the vehicle at **(A)** 0.5 and **(B)** 1 h. Values are mean ± SEM (*n* = 9), **P < 0.01. **(C)** Cell necrosis (measured by LDH release) in porcine monocyte-derived macrophages incubated with control (10% FBS in IMDM), tylvalosin (0.1, 1, or 10 μg/mL), or 1% Triton X-100 (positive control) for 0.5–24 h. Values were expressed as a fold change compared as a ratio to control. Values among controls and tylvalosin-treated cells did not differ among each other (*n* = 6–9/group). Data are mean ± SEM, *P < 0.05 versus control.

At the times when tylvalosin induced apoptosis in neutrophils (0.5 and 1 h; Figure 1) or in monocyte-derived porcine macrophages (12 and 24 h; Figure 2), the antibiotic did not alter levels of necrosis in these cells as detected by release of lactate dehydrogenase (LDH) (Figure 3).

### Tylvalosin Promotes Efferocytosis of Porcine Neutrophils by Macrophages, Without Altering Phagocytosis

**Figure 4:**
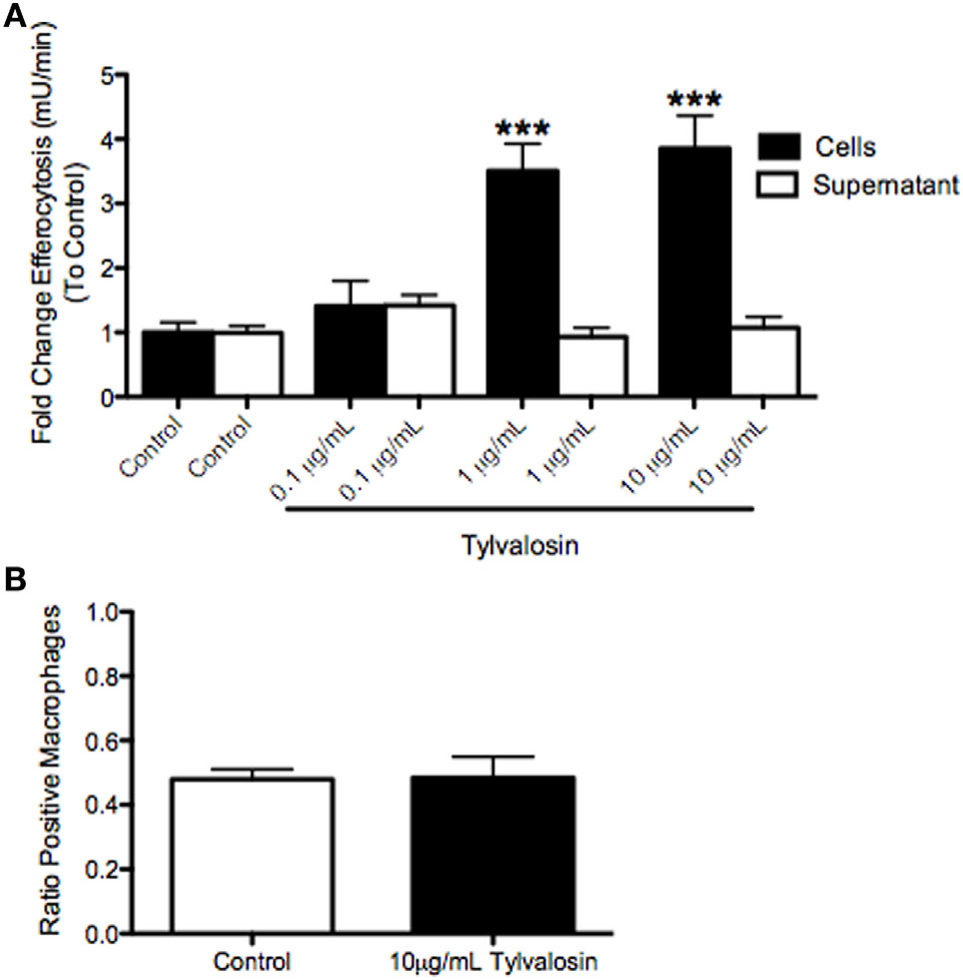
Tylvalosin promotes efferocytosis, but does not modulate mannose-dependent phagocytosis in porcine monocyte-derived macrophages *in vitro*. **(A)** Myeloperoxidase (MPO), a marker for neutrophils, was found at increasing concentrations in macrophages given neutrophils treated with tylvalosin for 0.5 h. Porcine macrophages were treated for 2 h with untreated neutrophils (control; 10% FBS in HBSS for 0.5 h) and PMNs treated with 0.1, 1, or 10 μg/mL tylvalosin for 0.5 h. Values are expressed as a fold change to control of MPO activity measured in mU/min. Data are mean ± SEM (*n* = 3/group). ***P < 0.001 versus control. **(B)** Mannose-dependent phagocytic activity of zymosan particles is not changed in porcine macrophages that show increased efferocytosis at 2 h. Monocyte-derived macrophages were incubated with 1 mg/mL of zymosan particles in control media (10% FBS in HBSS) or 10 μg/mL TVN in IMDM for (B) 2 h (*n* = 6/group). Macrophages containing one or more ZYM (zymosan) particles were counted as “positive cells,” and values were calculated as a ratio of positive macrophages versus total macrophages. Data are means ± SEM.

To determine whether induction of tylvalosin-induced neutrophil apoptosis also lead to efferocytosis, porcine neutrophils were incubated with tylvalosin (0.1, 1.0, or 10 µg/mL) or control vehicle for 0.5 h as above and then coincubated with monocyte-derived macrophages for 2 h. Tylvalosin significantly increased levels of efferocytosis, in a concentration-dependent manner (Figure 4A). Induction of macrophage efferocytosis did not coincide with altered macrophage phagocytic activity (measured by uptake of zymosan particles), demonstrating that the tylvalosin-induced activation of substrate ingestion by macrophages was selective, at least in part, for apoptotic bodies (Figure 4B).

### Tylvalosin Inhibits the Production of Pro-Inflammatory LTB4 in Neutrophils

**Figure 5:**
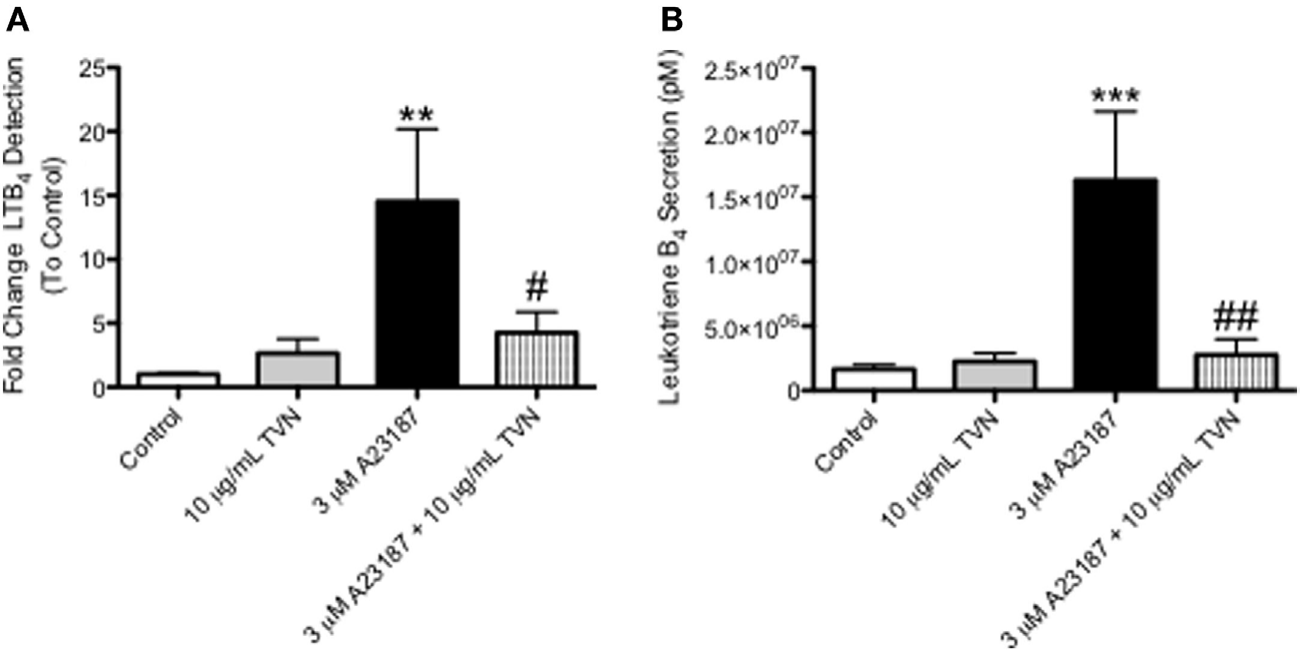
Tylvalosin inhibits the production of the pro-inflammatory lipid mediator LTB_4_, in calcium ionophore-activated porcine neutrophils. **(A)** Secreted levels of LTB_4_ from porcine neutrophils treated with 10% HI-FBS in HBSS (control) or 10 μg/mL TVN in the presence or absence of 3 μM calcium ionophore A23187 for 0.5 h were measured using reverse phase- high performance liquid chromatography (RP-HPLC). Values represent fold change of LTB_4_ peak integration values generated from arbitrary units on sample specific chromatograms where intensity correlated to LTB_4_ concentration. Data represent mean ± SEM (*n* = 7–10/group). **P < 0.01 versus unstimulated control; ^#^*P* < 0.05 versus 3 μM A23187. **(B)** Porcine neutrophils underwent these same treatments for 0.5 h and LTB_4_ release was measured using liquid chromatography-mass spectrometry (LC/MS). Values represent LTB_4_ levels in pM calculated from standard curves generated by integrating the area under the curve of elution peaks where intensity correlated to known LTB_4_ concentrations. Data represent mean ± SEM (*n* = 10–15/group). ***P < 0.001 versus unstimulated control; ^##^*P* < 0.01 versus 3 μM A23187.

Tylvalosin inhibited the production of LTB_4_ in calcium ionophore-activated neutrophils, as measured by ultra high performance liquid chromatography mass spectrometry (UHPLC-MS) (Figure 5, and Figure S1 in Supplementary Material). The activation by calcium ionophore is meant to reflect the high degree of neutrophil activation, and subsequent elevated levels of LTB_4_, synthesis known to occur in inflamed tissues.

### Tylvalosin Inhibits the Release of Pro-Inflammatory CXCL-8 and IL-1α in LPS-Stimulated Macrophages

**Figure 6:**
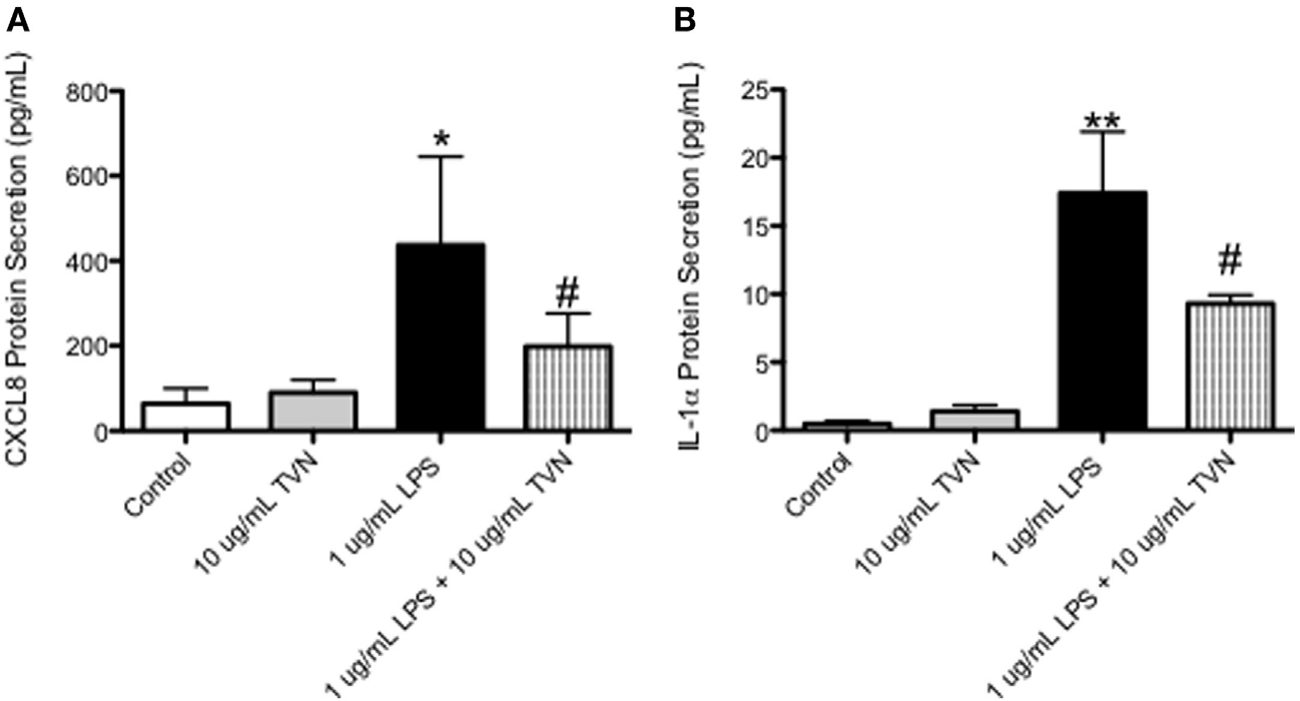
Tylvalosin treatment inhibits CXCL8 and IL-1α protein secretion in LPS-stimulated macrophages. Porcine macrophages were treated with control vehicle (10% HI-FBS in IMDM) or tylvalosin (10 μg/mL), in the presence of absence of LPS (1 μg/mL) for 2 h. Supernatants were collected and submitted to multiplex analysis of secreted chemokines/cytokines. Using this bead-based fluorescent assay and the standard curves it generates secreted protein was measured and values are expressed as picograms/milliliter. CXCL8 **(A)** and IL-1α **(B)** protein secretion was inhibited by tylvalosin in LPS-stimulated macrophages. Data represent mean ± SEM (*n* = 3–4/group). *P < 0.05, **P < 0.01 versus unstimulated control; ^#^*P* < 0.05 versus LPS-stimulated macrophages.

CXCL-8 (Figure 6A) and IL-1α (Figure 6B) production by LPS-stimulated macrophages was significantly lower in tylvalosin-treated cells, versus LPS-stimulated cells given vehicle. Porcine macrophages incubated with control vehicle or tylvalosin (10 µg/mL), in the presence of absence of LPS (1 µg/mL) for 2 h, were assessed for CXCL-8 and IL-1α production using a bead-based fluorescent multiplex system.

### Tylvalosin Stimulates the Release of Pro-Resolving LXA_4_ and RvD_1_ in Neutrophils

**Figure 7:**
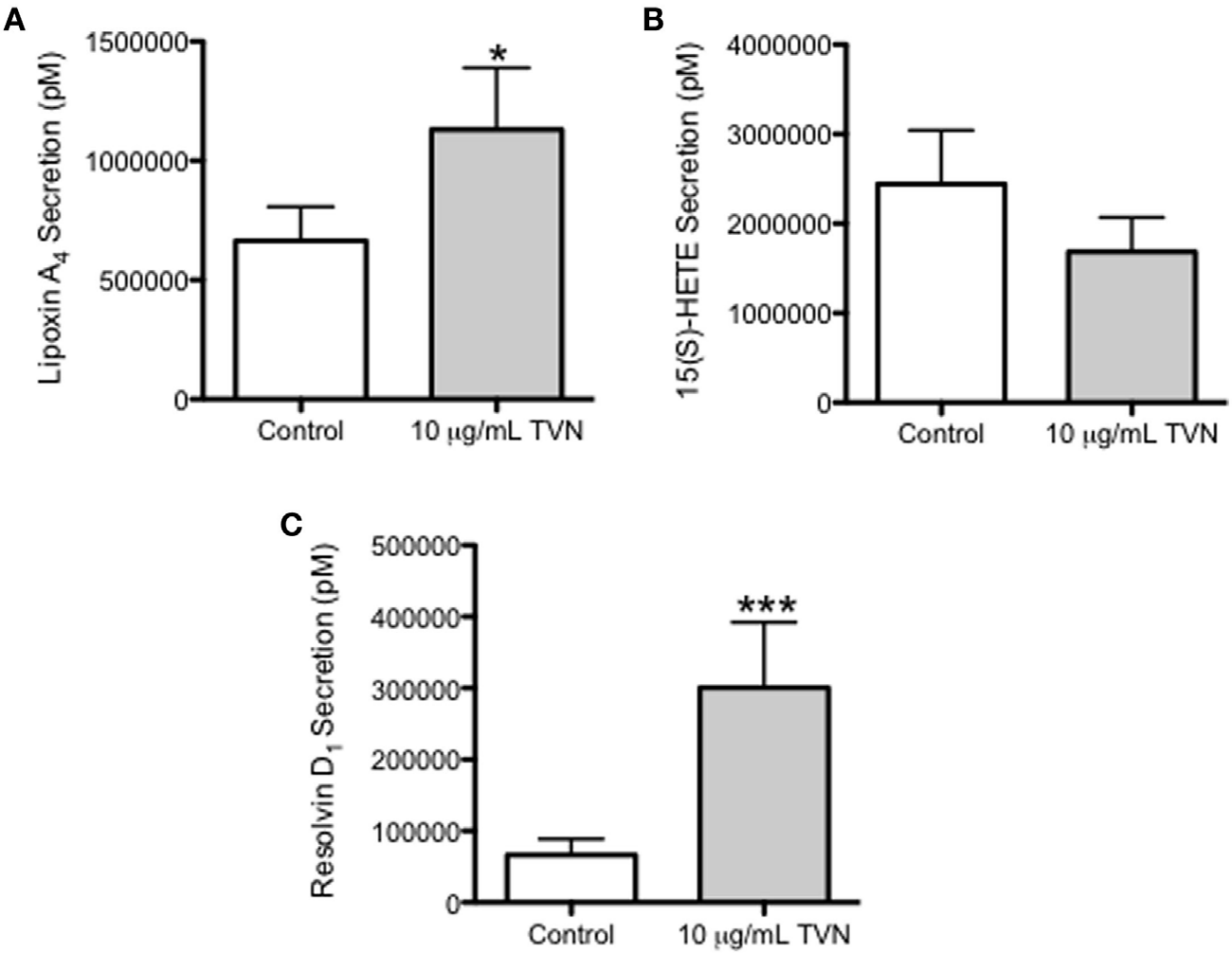
Tylvalosin promotes the secretion of the pro-resolution lipid mediators LXA_4_ and RvD_1_. Secreted levels of LXA_4_ **(A)**, its metabolite precursor 15(S)-HETE **(B)**, and RvD_1_ **(C)** were measured from porcine neutrophils treated with 10% HI-FBS in HBSS (control) or with 10 μg/mL tylvalosin for 0.5 h using liquid chromatography–mass spectrometry (LC/MS). Values represent mediator levels in pM calculated from standard curves generated by integrating the area under the curve of elution peaks where intensity correlated with known LXA_4_, 15(S)-HETE, or RvD_1_ concentrations, respectively. Data represent mean ± SEM (*n* = 18–20). *P < 0.05, ***P < 0.01 versus unstimulated control.

Porcine neutrophils incubated with control vehicle or tylvalosin were assessed for the production of LXA_4_ and its precursor 15(S)-hydroxyeicosatetraenoic acid (15(S)-HETE), and for RvD_1_, using LC/MS. Tylvalosin induced the release of LXA_4_ (Figure 7A, and Figure S1 in Supplementary Material) and RvD_1_ (Figure 7C). The effect of tylvalosin on 15(s) HETE production failed to reach statistical significance (Figure 7B).

### Tylvalosin Upregulates Cytosolic Phospholipase A_2_ (cPLA_2_) and Phospholipase C (PLC) Activity in Porcine Neutrophils

**Figure 8:**
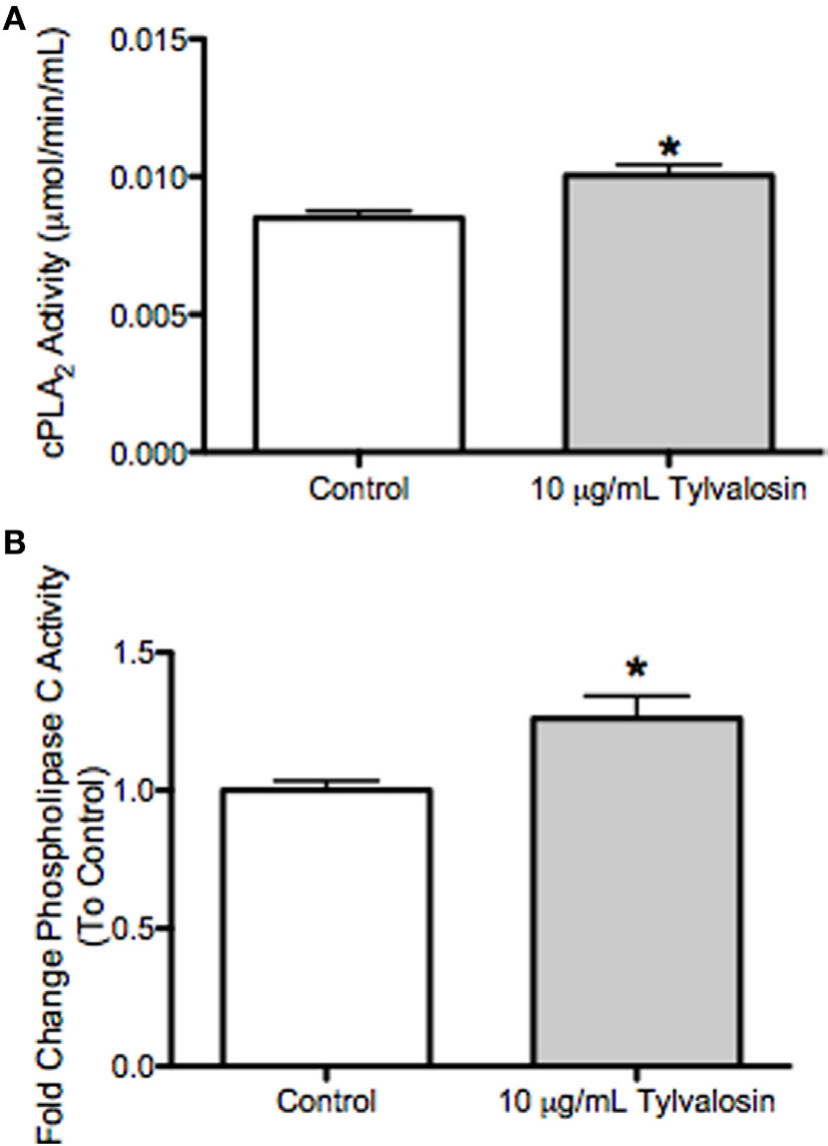
Tylvalosin increases intracellular phospholipase C (PLC) activity in porcine neutrophils. **(A)** Activity of cytosolic phospholipase A_2_ (cPLA_2_) was not different in cell lysates treated with tylvalosin (10 μg/mL for 0.5 h) versus control cells given 10% HI-FBS in HBSS. Values represent cPLA_2_ enzymatic activity in μmol/min/mL. Data represent mean ± SEM (n = 5/group). *P < 0.05 versus control. **(B)** Activity of intracellular PLC was significantly increased in cell lysates treated with tylvalosin (10 μg/mL for 0.5 h) versus Control cells given 10% HI-FBS in HBSS. Values represent enzymatic activity for PLC as a fold change ratio to control. Data represent mean ± SEM (*n* = 4/group). *P < 0.05 versus control.

In an attempt to gain further mechanistic insight into the modulation of neutrophil lipid mediator release by porcine neutrophils exposed to tylvalosin, additional experiments assessed effects on cytosolic phospholipase A_2_ (cPLA_2_) and PLC activity. cPLA_2_ can release arachidonate from membrane phospholipids; and arachidonate can be generated by diacylglycerol lipase metabolism of diacyglycerol (45), which along with inositol trisphosphate can be generated by PLC from membrane phospholipids. This generation of arachidonic acid would permit the synthesis of lipid mediators from the released arachidonic acid (20). In porcine neutrophils incubated with tylvalosin, the activity of cPLA_2_ and PLC were significantly elevated compared to vehicle-treated controls (Figure 8).

## Discussion

Treatments that offer dual antimicrobial and anti-inflammatory/pro-resolution actions may yield superior clinical benefits during bacterial infections (13, 32–34). The present study, using a model of tylvalosin-induced immunomodulation in porcine neutrophils and macrophages, reveals a novel antibiotic-induced release of the potent pro-resolution mediators RvD_1_ and LXA_4_ by neutrophils. The effects were associated with activation of PLC. Tylvalosin was found to inhibit the production of pro-inflammatory mediators, such as LTB_4_ in neutrophils, and CXCL-8 and IL-1α in LPS-stimulated macrophages. Finally tylvalosin induced apoptosis in neutrophils and monocyte-derived macrophages in concentration- and time-dependent fashion and promoted efferocytosis. The effects appeared to be at least in part drug-selective as lincomycin, another antibiotic used to treat porcine pneumonia, did not generate these effects. Together, the findings demonstrate that tylvalosin, independently of its antimicrobial benefits, has potent immune-modulating properties that carry great potential for the resolution of inflammation *in vivo*, a potential that warrants further investigation.

Pathogenesis and severe tissue damage in porcine bacterial pneumonia result at least in part from local cell necrosis and self-amplifying recruitment of neutrophils, as it does during a variety of inflammatory diseases in animals and humans (3, 4, 9, 12, 13). The containment of these dying cells, and their removal by apoptosis and efferocytosis, represent critical stages of the resolution of inflammation (15, 46). Under homeostatic conditions, neutrophils are inactivated and removed from the site of inflammation by apoptosis (programmed cell death). During apoptosis, the destruction of intracellular organelles occurs while cell plasma membrane integrity is preserved, hence preventing the release of pro-inflammatory and histolytic compounds into the surrounding milieu. In this context, therefore, promotion of leukocyte apoptosis has been suggested to confer potent pro-resolution properties to a drug as this physiologically regulated process does not appear to interfere with the antimicrobial function of the cells (12, 13, 18, 22, 47). Consistent with recent observations that found that cyclin-dependent kinase inhibitors as well as veterinary macrolides, such as tilmicosin and tulathromycin, enhance the resolution of inflammation by inducing neutrophil apoptosis, the present study is based on the hypothesis that antibiotics that promote inflammatory cell apoptosis may have significant therapeutic benefits in infectious diseases in which host inflammation is central to pathogenesis (12, 13, 37, 38, 47). Recent observations indicate that tylvalosin may have immune-modulating effects in a murine model of acute lung injury, as well as in piglets challenged with PRRSV, by suppressing the activation of NF-κB (42). These findings possibly point to an intracellular mechanism associated with the high affinity for uptake of this compound inside leukocytes. This hypothesis is consistent with the recent observation that intracellular accumulation of tulathromycin and other macrolides may inhibit intracellular phospholipases, which are known to regulate lipid mediator synthesis as well as phospholipidosis-dependent cell apoptosis (23, 34). Indeed, tylvalosin has been found to reduce the expression of phospholipase A_2_ (42). In contrast, the present findings suggest that tylvalosin may increase cytosolic PLC and phospholipase A_2_ in porcine neutrophils. Further studies are needed to determine whether this discrepancy may reflect different biological effects in different model systems. In mice or piglets with pulmonary inflammation, tylvalosin provides clinical benefits and attenuates inflammatory injury in association with markedly reduced levels of pro-inflammatory cytokines, such as CXCL-8 (IL-8), IL-6, and IL-1beta (42). Findings from the present study demonstrate that tylvalosin, but not lincomycin, induces caspase-3-dependent apoptosis in porcine neutrophils. Tylvalosin also has a delayed pro-apoptotic effect in porcine macrophages, and the induction of apoptosis in both cell types did not coincide with heightened necrosis. The pro-apoptotic effects of tylvalosin also lead to increased efferocytosis, without any concurrent alteration to the mannose-dependent phagocytosis by macrophages of zymosan particles. These effects are consistent with observations made for other macrolides, including azithromycin, tilmicosin, and tulathromycin, but not with non macrolide antibiotics (12, 23, 34, 37, 38, 48, 49). The mechanisms responsible for such a drug-selective effect remain unclear, but may possibly be linked to the modulation of cytosolic phospholipases associated with the preferential intracellular uptake of the drug (34, 40, 50).

Macrolides may modulate host cytokine responses and yield angiogenic effects, as well as anticancer properties, in addition to their antimicrobial effects (35, 51, 52). Consistent with these observations, the findings reported here found that tylvalosin inhibits pro-inflammatory IL-1α and the chemokine CXCL-8 in macrophages. Less is known about how these drugs may affect the production of lipid mediators. LTB_4_, like CXCL-8, is a powerful neutrophil chemoattractant implicated in the pathogenesis of pneumonia (12, 34, 53). LTB_4_ is also responsible for neutrophil degranulation, local release of superoxide radicals and elastase, and along with β-integrins, causes mucosal swarming phenotypes, which further perpetuate leukocyte recruitment and inflammatory injury (20, 22, 53–55). Results presented here demonstrate that tylvalosin is able to directly inhibit LTB_4_ release from activated porcine neutrophils, independently of its antimicrobial properties.

Using UHPLC-MS, the present study identified a novel antibiotic effect that triggers the production of potent pro-resolution lipid metabolites, RvD_1_, and LXA_4_. We recently reported that tulathromycin was able to promote the production of LXA_4_ by porcine neutrophils (34). The clinical significance of these pro-resolution lipid mediators is underscored by ongoing human clinical trials using LXA_4_ for gingivitis treatment, type 2 diabetes mellitus, atherosclerosis, and asthma (46, 56). Furthermore, in view of its concurrent inhibition of LTB_4_, tylvalosin may offer a powerful tool to investigate antibiotic-induced lipid mediator class switching, which is of critical importance to the resolution of inflammation (14, 55). Indeed, lipid mediator class switching can be prompted *via* efferocytosis. In this process, neutrophils undergo changes at the gene expression level through the action of prostaglandins E_2_ and D_2_, which can decrease neutrophil leukotriene generation and switch arachidonic acid metabolism by increasing expression of 15-lipoxygenase to generate lipoxins and D-series resolvins (55, 56). Further research is required to determine whether tylvalosin alters the transcription of enzymes involved in lipid mediator class switching, or whether it may act on allosteric sites to inhibit or stimulate these enzymes. The present studies remain observations from experiments *in vitro*, which now warrant confirmation using a model of live infection in piglets. This response to tylvalosin may pave the road toward the characterization of novel pro-resolution properties of macrolides and other drugs. Whether and how in turn these may play in the context of antimicrobial resistance also represent an important are for future research.

## Materials and Methods

### Animals

Healthy large white/Landrace cross 10- to 12-week-old female or castrated male pigs weighing an average of 32.5 ± 2.5 kg were used, as validated previously (12). Pigs were given a single injection of EXCEDE© 100 for Swine (ceftiofur crystalline free acid; Zoetis, Parsippany, NJ, USA) upon arrival as prophylaxis against bacterial septicemic disease following relocation stress. After a minimum of 1-week acclimation, animals were bled once every 2 weeks, except in the case of co-incubation studies where cells from the same pigs were required, and the same pig was bled once per week for two consecutive weeks. Pigs were kept for 10 ± 2 weeks. Animals were euthanized and tissues made available for secondary teaching and research use. Pigs were euthanized by intracardiac injection with sodium pentobarbital in accordance with the standards of the Canadian Council on Animal Care.

All care and experimental practices were conducted under the guidelines of the Canadian Council on Animal Care and approved by the University of Calgary Life and Environmental Science Animal Care Committee.

### Blood Collection

Blood was drawn from the cranial vena cava of restrained animals (<60 kg BW) or the jugular vein (>60 kg BW) into acid citrate dextrose vials (ACD solution; BD Vacutainer Systems 364606). Immediately following each collection, pigs were given the NSAID meloxicam (0.4 mg/kg; Boehringer Ingleheim, Burlington, ON, Canada), to provide analgesia.

### Monocyte Isolation and Macrophage Differentiation

Cell preparations were performed as validated previously (12, 48). Briefly, blood samples were pooled into polypropylene conical tubes (BD Falcon) and centrifuged at 1,200 *g* for 20 min at 4°C in a Thermo Scientific Heraeus Megafuge 16R (Thermo Scientific, Waltham, MA, USA) without braking to allow for plasma separation. The buffy coat was removed and added to a clean polypropylene conical tube and diluted 1:1 with cold filter-sterilized 0.9% NaCl solution. A 5 mL of polysucrose and sodium diatrizoate gradient (Histopaque-1077; Sigma-Aldrich) was overlaid onto the mixture and centrifuged at 1,500 *g* for 40 min at 4°C without breaking. Histopaque-1077 causes erythrocytes and granulocytes to sediment, while monocytes remain at the saline/Histopaque-1077 interface. Monocytes were resuspended (1 × 10^6^ cells/ml) in Iscove’s Modified Dulbecco’s medium (IMDM; Thermo Fisher 12440) containing 10% heat-inactivated fetal bovine serum (HI-FBS; Life Technologies 12484028). Cells were counted using a hemocytometer and cell viability was assessed with 0.1% trypan blue staining (Flow Laboratories, Inc.). Differential cell counts were performed on cytospin preparations stained with Diff-Quik (Baxter Healthcare Corp., Miami, FL, USA) preparation according to the manufacturer’s instructions.

Monocytes were incubated with IMDM containing 10% HI-FBS in a humidified 37°C and 5% CO_2_ incubator, non-adherent cells were removed, and the medium was replaced with IMDM (Thermo Fisher 12440) containing 10% HI-FBS (Life Technologies 12484028), 100 U/mL pencillin and 100 U/mL streptomycin (both from Sigma-Aldrich). Adherent monocytes were incubated for 7 days to allow for macrophage differentiation. Experiments were performed using IMDM containing 10% HI-FBS. Macrophage differentiation (>95%) was confirmed by microscopic morphological evaluation using Diff-Quik stain (Baxter Healthcare Corp., Miami, FL, USA).

### Neutrophil Isolation

Neutrophils were isolated as previously (12, 48). Blood was centrifuged at 1,200 *g* for 20 min at 4°C. The plasma and buffy coat were removed and the remaining cells were washed with HBSS and spun again at 1,200 *g* at 4°C for 10 min without breaking. Contaminating erythrocytes were eliminated with a cold filter-sterilized hypotonic lysis solution (10.6 mM Na_2_HPO_4_, 2.7 mM NaH_2_PO_4_) for 1 min. Isotonicity was then restored by adding the same volume of cold filter-sterilized hypertonic restoring solution (10.6 mM Na_2_HPO_4_, 2.7 mM NaH_2_PO_4_, 462 mM NaCl). The cell mixture was centrifuged at 1,200 *g* for 10 min at 4°C without breaking, and the supernatant was discarded. Cells were resuspended in 37°C HBSS (Sigma) containing 10% HI-FBS (Thermo Fisher). Concentrations of neutrophils and percentage of viable cells were evaluated using 0.1% trypan blue staining (Flow Laboratories Inc., McLean, VA, USA), in a hemocytometer (VWR Scientific; Invitrogen). Differential cell counts were performed on cytospin preparations (CytoSpin 4 Cytocentrifuge, Thermo Scientific) stained with Diff quick (Diff-Quik, Baxter Healthcare Corp., Miami, FL, USA). Neutrophils were >90% pure and >90% viable.

### Detection of Apoptotic Cell Death, Using Terminal Deoxynucleotidyltransferase-Mediated dUTP-Biotin Nick End Labeling (TUNEL)

Apoptotic cell death was assessed using *in situ* terminal deoxynucleotidyltransferase-mediated uridine 5′ triphosphate-biotin dUTP nick end labeling (TUNEL) on cytopsin preparations, as previously described (12) (Roche Applied Science). This enzymatic system labels DNA breaks at the free 3′OH terminus with nucleotides conjugated fluorescein. Cytospin and chamber slide preparations were fixed using 4% paraformaldehyde in PBS (Santa Cruz Biotechnology) and permeabilized using 0.1% Triton-X-100 (Sigma-Aldrich) in 0.1% sodium citrate. Slides were subsequently washed with PBS and incubated with the TUNEL reaction at 37°C at 1 h in a dark, humidified chamber according to the manufacturer’s instructions (Roche Applied Science). Slides were counterstained with DAPI (Sigma-Aldrich).

Slides were untreated (control), tylvalosin treated (0.1, 1.0, or 10 µg/mL; 0.096–9.6 µM), lincomysin treated (11.3 µM), or staurosporine (1 µM) treated. High tylvalosin concentrations preferentially accumulate in leukocytes, an effect recently associated with anti-inflammatory properties that have been associated with the suppression of NF-κB activation in animals treated *in vivo* (57). The concentrations used here are consistent with those, based on the same rationale, used and validated in previous studies using macrolides like tilmicosin and tulathromycin as well as tylvalosin, which all have elevated intra-leukocyte accumulation properties (12, 13, 23, 34). Tylvalosin (aivlosin) obtained from ECO Animal Health (London) was used as a powder diluted to the appropriate concentrations in sterile LPS-free HBSS (Sigma).

Images were taken at 400× magnification using a Leica DMR fluorescent microscope with Retiga 2000X (Q Imaging, Surrey, BC, Canada) and Q Capture Suite software (Q Imagining, Surrey, BC, Canada). Images were analyzed using Image J software (National Institute for Health, Bethesda, MD, USA).

### Detection of Apoptotic Cell Death, Using a Cell Death ELISA

Quantities of apoptotic mono- and oglionucleosomes were determined in triplicates using a cell death detection ELISA as previously (12, 37, 48) (ELISA; Roche Molecular Biochemical, Laval, QC, Canada; 11544675001). Neutrophils (6 × 10^6^) and macrophages (1 × 10^6^) were incubated in vehicle medium supplemented with 10% HI-FBS (HBSS or IMDM, respectively), tylvalosin (0.1, 1.0, or 10 µg/mL; 0.096–9.6 µM) or equimolar concentrations of lincomycin (11.3 µM), a non macrolide antibiotic also used to treat porcine pneumonia, at 37°C and 5% CO_2_ for 0.5–24 h. Cells treated with staurosporine (1 µM) served as a positive controls.

### Cleaved Caspase-3 Activity

Caspase-3 activity in monocyte-derived macrophages (on Day 7 of differentiation) in tissue culture plates (1 × 10^5^ cells/mL) was measured using a caspase-3 fluorescent activity assay (FITC-DEVE-FMK; EMD Millipore QIA70 La Jolla, CA, USA) in cells treated with IMDM with either 10% HI-FBS vehicle (control), tylvalosin (0.1, 1, 10 µg/mL), lincomycin (11.3 µM), or staurosporine (1 µM) at 37°C and 5% CO_2_ for 0.5–24 h, as per the manufacturer’s instructions. Fluorescence was measured with a SpectraMax M2e microplate reader (Molecular Devices).

### Western Blotting for Cleaved Capsase-3

Cleaved caspase-3 in monocytes and macrophages was also measured using Western blotting, as previously (38). Treated cells were washed with HBSS and lysed using a RIPA lysis buffer containing a protease inhibitor pellet (Complete Mini; Roche Diagnostics). Total protein concentration was determined using a Bradford protein assay (Bio-Rad Laboratories, Mississauga, ON, Canada). Proteins were resolved on 10% SDS-polyacrylamide gel, and membranes were blocked in 5% Bovine Serum Albumin in TBS containing 0.5% Tween 20 (TBS-T). Membranes were exposed to rabbit anti-cleaved caspase-3 antibodies (Cell Signaling Technology, Danvers, MA, USA) at a dilution of 1:500 and incubated with anti-rabbit secondary antibodies (Cell Signaling) at a dilution of 1:1,000. Samples were visualized using chemiluminescence (ECL plus; VWR) with a Chemidoc (Bio-Rad).

### Assessment of Cell Necrosis

Cells (1 × 10^5^ cells/mL) were treated with vehicle medium alone (control), tylvalosin (0.1, 1.0, or 10 µg/mL), lincomycin (11.3 µM), and 1% Triton-X 100 in media (positive control) and supernatants were assayed for levels of LDH using a cytotoxicity detection kit (Roche Applied Science). Plates were read on a SpectraMax M2e (Molecular Devices) at 492 nm.

### Detection of Efferocytosis

To quantify macrophage efferocytosis of apoptotic neutrophils, polymorphonuclear cells (1 × 10^4^ cells/mL) were incubated with either control medium (10% HI-FBS in HBSS), tylvalosin (0.1, 1.0, or 10 µg/mL), lincomycin (11.3 µM), or the positive control for apoptosis staurosporine (1 µM), for 30 min (23). Following treatment, cells were washed with HBSS (free of serum), centrifuged at 850 *g* for 5 min and resuspended in warm IMDM containing 10% HI-FBS and cocultured with monocyte-derived macrophages for 2 h at 37°C in a humidified incubator. Both the supernatant fraction containing free neutrophils, and the coculture monolayer fraction, were lysed with lysis buffer (1:1 ratio of 1 M citrate and 10% Triton-X 100; Sigma-Aldrich) and incubated for 15 min at 4°C while shaking. Myeloperoxidase activity, as a marker of neutrophil accumulation, was measured in coculture supernantants and monolayers with a kinetic assay, as described previously (12). Immediately before reading the kinetic absorbance, *o*-dianisidine reagent (Sigma) was added. Absorbance readings were taken at 460 nm once every 30 s for 16 min using a SpectraMax M2e microplate reader (Molecular Devices). Enzyme activity was defined as the change in optical density over time (mU/min).

### Macrophage Phagocytosis of Zymosan Particles

Mannose-dependent phagocytic uptake of zymosan A particles from *Saccharomyces cerevisiae* (β-glucan of yeast cell wall; Sigma) by mature macrophages was measured as previously (23). Mature macrophages were treated with 10% HI-FBS in IMDM (control), or 10 µg/mL TYL, for 2 h, a concentration at which tylvalosin significantly increased apoptosis and efferocytosis. Treated macrophages were exposed to zymosan (10 mg/mL) for 2 h. Cells were stained with Diff-Quik, and zymosan particles engulfed inside macrophages were enumerated. Macrophages containing one or more ZYM particles were counted as “positive cells” and values were calculated as a ratio of positive macrophages versus total macrophages.

### Measurements of Leukotriene B4 Using Reverse-Phase High-Performance Liquid Chromatography (RP-HPLC)

Supernatants were collected from porcine neutrophils incubated with HBSS or 10 µg/mL tylvalosin in the presence or absence of 3 µM A2318, a calcium ionophore stimulating LTB_4_ synthesis as does an inflamed environment, for RP-HPLC analysis to detect leukotriene B_4_ (LTB_4_) as validated previously (12, 34). Cell supernatants were collected and evaporated under a gentle stream of nitrogen gas. Samples were then reconstituted in 1 mL of 25% acetonitrile in water and loaded onto a C_18_ column. Sample elution was achieved using a gradient of 25% acetonitrile–0.1% trifluoroacetic acid (TFA)–H_2_O to 65% acetonitrile–0.1% TFA–H_2_O (flow rate of 1 mL/min). An initial RP-HPLC run was done to identify retention time of the LTB_4_ peak, which was determined by UV profiling of a commercial LTB^4^ standard (Cayman Chemical 20110) at 270 nm with elution between 36–37 min. This approach has been validated previously (12, 34).

### Measurements of CXCL-8 and IL-1α

Supernatants of macrophages treated with vehicle (control), or 10 µg/mL tylvalosin, in the presence or absence of LPS (1 µg/mL from *E. coli* 0111:B4; Sigma-Aldrich), for 2 h at 37°C and 5% CO_2_ were collected and stored at −70°C until further analysis. Samples were submitted to multiplex bead-based analysis for cytokine/chemokine biomarkers (Eve Technologies, Calgary, AB, Canada).

### Measurements of Lipid Mediators Using Ultra High Performance Liquid Chromatography Mass Spectrometry (UHPLC-MS)

Supernatants collected from porcine leukocytes incubated with HBSS or 10 µg/mL tylvalosin in the presence or absence of 3 µM A23187 for 30 min. The supernatants were directly transferred to 1 mL of acidified ddH_2_0 to allow protein precipitation and then supplemented with 2 mL of anhydrous ethyl acetate (Sigma-Aldrich). The tubes were mixed for 30 min at 4°C and centrifuged at 1,200 *g* for 10 min. The upper organic phase was transferred into a new glass tube, while a second liquid–liquid extraction was performed on the remaining aqueous layer. The upper organic layers were pooled and evaporated under nitrogen gas at room temperature. Lipid extracts were dissolved in 200 µL of ice-methanol (HPLC-grade; Fisher Scientific) and stored at −70°C for UHPLC-MS analysis. Samples were analyzed on a TSQ Quantum Access Max Triple Quadrupole mass spectrometer in negative ion mode using previously established SRM transitions (335.2 to >195.1 *m/Z* at 16 eV for leukotriene B4; 351.2–115.0 at 19 eV for lipoxin A4). Separation of metabolites was achieved using a Thermo Fisher Scientific Vanquish UHPLC platform using an Accucore™ Polar Premium UHPLC column (2.6 μM × 150 mm × 2.1 mm; Thermo Fisher 28026-152130). A binary solvent system comprised of 0.1% formic acid in HPLC-grade water (Solvent A) and 0.1% formic acid in HPLC-grade acetonitrile (Solvent B) was used. A 14.5-min linear gradient starting from 3% solvent A and 97% solvent B and ending with 20% Solvent A and 80% Solvent B was used to elute compounds. Elution of tested compounds was determined by UHPLC-MS profiling of commercial standards (Cayman Chemical).

### Detection of Phospholipase Activity

Cytosolic phospholipase A_2_ (cPLA_2_) was measured in porcine neutrophils treated with 10% HI-FBS in HBSS (control) or tylvalosin (10 µg/mL; 9.6 µM) for 30 min, using a cytosolic phospholipase A_2_ assay (Cayman Chemical 765021). Briefly, porcine neutrophils were treated with 10% HI-FBS in HBSS (control) or tylvalosin (10 µg/mL) for 30 min. Following incubation, the cells were centrifuged at 850 *g* for 5 min at 4°C, pellets were washed with HBSS, resuspended in 1 mL of cold buffer (50 mM HEPES pH = 7.4 containing 1 mM EDTA), and sonicated at level 3 for 5 s. Cell sonicates (550 Sonic Dismembrator, Fischer Scientific) were stored until further analysis at −70°C. PLC activity was measured in porcine neutrophils treated as above using the EnzChek Direct PLC assay (Molecular Probes E10215). Colorimetric changes were measured at 405 nm with a SpectraMax M2e microplate reader (Molecular Devices).

### Statistical Analyses

Data were expressed as means ± SEM. Statistical analyses were performed using Prism 5 software. Results were compared using one way analysis of variance followed by a Student’s *t*-test, or a Dunnett’s multiple comparison *post hoc* test to compare between control and treatment groups, and/or Tukey’s multiple comparison *post hoc* test. Using multiple replicates of a minimum of three separate, independent experiments, *P* values < 0.05 were considered statistically significant.

## Ethics Statement

This study was carried out in accordance with the recommendations of Canadian Council on Animal Care and approved by the University of Calgary Life and Environmental Science Animal Care Committee. The protocol was approved by the University of Calgary Life and Environmental Science Animal Care Committee.

## Author Contributions

RM collected, isolated, and purified leukocytes for experiments, continuing to perform the majority of experiments, literature searches, and the writing of the manuscript. DL also aided in the collection and isolation of leukocytes, with some macrophages experiments, and completed most of the LC/MS analyses. SS performed some of the initial neutrophil experiments. BR and MH aided in the development of the RP-HPLC protocols and the running of those samples. GM aided in the weekly blood collection from the animals as well with the care of the animals and their housing. AB was the principal investigator of this study. It was from him that the initial proposal to begin this study began and it was through his guidance and hard work that the study and manuscript were able to come to fruition.

## Conflict of Interest Statement

EA is an employee of ECO Animal Health. The other authors declare that the research was conducted in the absence of any commercial or financial relationships that could be construed as a potential conflict of interest.
