# Supplementary material for: Anti-Inflammatory Benefits of Antibiotics: Tylvalosin Induces Apoptosis of Porcine Neutrophils and Macrophages, Promotes Efferocytosis, and Inhibits Pro-Inflammatory CXCL-8, IL1α, and LTB4 Production, While Inducing the Release of Pro-Resolving Lipoxin A4 and Resolvin D1
Source: Front Vet Sci. 2018 Apr 11;5:57. doi: 10.3389/fvets.2018.00057 (PMC5905233; doi:10.3389/fvets.2018.00057)
Supplement: Supplementary file 1 [file data_sheet_1.docx]

Supplementary figure 1:

Representative extracted ion chromatograms illustrating the opposite effects of tylvaloin on pro-inflammatory LTB_4_ and pro-resolution LXA_4_. Tylvalosin reduces the production of leukotriene B4 in calcium ionophore stimulated neutrophils while it increases lipoxin A_4_ in neutrophils. **A.** Extracted Ion Chromatogram of leukotriene B4 (LTB4) production by Neutrophils. Neutrophils (~5.0 x 10^7^ cells/ml) were incubated with tylvalosin (10 µg/ml) in presence or absence of calcium ionophore (3µM) for 30 minutes (upper panel). 100 nM of Leukotriene B4 was run as an external standard (lower panel) to verify the observed retention time. **B.** Extracted Ion Chromatogram of Lipoxin A4 production by Neutrophils (~5.0 x 10^7^ cells/ml) exposed to tylvalosin (10 µg/mL) for 30 minutes (upper panel). 100 nM of lipoxin A4 was added as an external standard (lower panel) to verify the observed retention time. Lipid mediators were isolated by liquid-liquid extraction and analyzed by UHPLC-MS using a TSQ Quantum Access Max Triple Quadrupole mass spectrometer coupled to a Vanquish UHPLC system (Thermo Fisher). Analysis was performed in negative ion mode using previously established SRM transitions (335.2->195.1m/Z at 16eV for leukotriene B4; 351.2-115.0 at 19eV for lipoxin A4).

A.





B.
